# Functional characterization of a novel arachidonic acid 12S‐lipoxygenase in the halotolerant bacterium *Myxococcus fulvus* exhibiting complex social living patterns

**DOI:** 10.1002/mbo3.775

**Published:** 2018-12-17

**Authors:** Kateryna Goloshchapova, Sabine Stehling, Dagmar Heydeck, Maximilian Blum, Hartmut Kuhn

**Affiliations:** ^1^ Institute of Biochemistry Charité—Universitätsmedizin Berlin, Corporate Member of Freie Universität Berlin, Humboldt‐Universität zu Berlin, and Berlin Institute of Health Berlin Germany; ^2^ Institute of Biology Humboldt University Berlin Berlin Germany

**Keywords:** eicosanoids, lipid metabolism, mutagenesis, oxidative stress, social behavior

## Abstract

Lipoxygenases are lipid peroxidizing enzymes, which frequently occur in higher plants and mammals. These enzymes are also expressed in lower multicellular organisms but here they are not widely distributed. In bacteria, lipoxygenases rarely occur and evaluation of the currently available bacterial genomes suggested that <0.5% of all sequenced bacterial species carry putative lipoxygenase genes. We recently rescreened the public bacterial genome databases for lipoxygenase‐like sequences and identified two novel lipoxygenase isoforms (*MF‐LOX1 and MF‐LOX2*) in the halotolerant *Myxococcus fulvus*. Both enzymes share a low degree of amino acid conservation with well‐characterized eukaryotic lipoxygenase isoforms but they involve the catalytically essential iron cluster. Here, we cloned the *MF‐LOX1* cDNA, expressed the corresponding enzyme as N‐terminal hexa‐his‐tag fusion protein, purified the recombinant enzyme to electrophoretic homogeneity, and characterized it with respect to its protein‐chemical and enzymatic properties. We found that *M. fulvus* expresses a catalytically active intracellular lipoxygenase that converts arachidonic acid and other polyunsaturated fatty acids enantioselectively to the corresponding n‐9 hydroperoxy derivatives. The enzyme prefers C_20_‐ and C_22_‐polyenoic fatty acids but does not exhibit significant membrane oxygenase activity. The possible biological relevance of *MF‐LOX1* will be discussed in the context of the suggested concepts of other bacterial lipoxygenases.

## 1 INTRODUCTION

Lipoxygenases (LOXs) form a heterogeneous family of fatty acid dioxygenases (Haeggstrom & Funk, 2011), which frequently occur in higher plants (Andreou & Feussner, 2009) and mammals (Kuhn, Banthiya, & Leyen, 2015) but have also been detected in lower organisms (Anterola et al., 2009; Hansen et al., 2013; Horn et al., 2015; Mortimer, Järving, Brash, Samel, & Järving, 2006; Yuan et al., 2014). In mammals, LOXs have been implicated in cell differentiation and maturation (Brash, Yu, Boeglin, & Schneider, 2007; Krieg & Furstenberger, 2014; Rapoport & Schewe, 1986; van Leyen, Duvoisin, Engelhardt, & Wiedmann, 1998) but also play important roles in human pathologies (Ackermann, Hofheinz, Zaiss, & Kronke, 2017; Chen, Sheller, Johnson, & Funk, 1994; Colakoglu, Tuncer, & Banerjee, 2018; Eckl et al., 2009; Haeggstrom & Funk, 2011; Harats et al., 2005; Kronke et al., 2009; Kuhn et al., 2015). In bacteria, LOXs have also been detected but here they occur at much lower frequency. A systematic search for putative LOX genes in the bacterial genomic sequences database (2013) revealed that the 3,700 deposited bacterial genomes (2013) involved 38 putative LOX genes (Hansen et al., 2013). Since certain bacterial species possess more than one LOX gene, it was concluded that <1% all bacterial species carry LOX genes. A more stringent search carried out in 2015 suggested that among the 13,000 bacterial genomes sequenced at this time some 60 species involved putative LOX genes (Horn et al., 2015). Although the vast majority of these potential bacterial LOXs has not been characterized, it was concluded that the presence of these enzymes may not be essential for bacterial life (Horn et al., 2015).

The first bacterial LOX was discovered in 1973 in the opportunistic human pathogen *Pseudomonas aeruginosa* (Shimahara & Hashixume, 1973)*.* This protein (PA‐LOX) was later on characterized as secreted arachidonic acid 15‐lipoxygenating enzyme (Banthiya et al., 2016; Garreta et al., 2013; Lu et al., 2013; Vance, Hong, Gronert, Serhan, & Mekalanos, 2004; Vidal‐Mas, Busquets, & Manresa, 2005). The crystal structure of PA‐LOX was solved at a molecular resolution of 1.4 Å (Banthiya et al., 2016; Garreta et al., 2013) and it differed from other pro‐ and eukaryotic LOX isoforms in two major aspects: (a) While the polypeptide chains of most eukaryotic LOX are folded into a two‐domain structure consisting of small N‐terminal β‐barrel domain and large helical catalytic domain (Choi, Chon, Kim, & Shin, 2008; Eek et al., 2012; Gilbert et al., 2011; Gillmor, Villasenor, Fletterick, Sigal, & Browner, 1997; Minor et al., 1996; Neau et al., 2009), the PA‐LOX polypeptide folds into a single domain structure (Banthiya et al., 2016; Garreta et al., 2013). (b) Recombinant PA‐LOX involves a bifurcated substrate‐binding pocket consisting of two hydrophobic cavities and a joining lobby. These internal cavities harbor a phosphatidylethanolamine molecule. The subcavity containing the sn1 fatty acid of the endogenous ligand involves the catalytic nonheme iron (Banthiya et al., 2016; Garreta et al., 2013). More recently, the crystal structure of a LOX isoforms from *Cyanothece *sp.* PCC 8801* was also solved (Newie et al., 2016). The biological activities of bacterial LOX have not been explored in detail. PA‐LOX has been implicated in pathogen–host interaction (Garreta et al., 2013) and in biofilm formation (Deschamps et al., 2016). More recently, the enzyme has been suggested as pathogenicity factor because of its capability of oxidizing membrane lipids of eukaryotic cells (Aldrovandi et al., 2018). A similar membrane lipid oxygenase activity has been suggested for a LOX isoforms from *Cyanothece *sp.* PCC 8801 *(Newie et al., 2016).

We recently rescreened the NCBI bacterial genome database and identified previously described LOX sequences in *Myxococcus xanthus* (WP_011551853.1, WP_011551854.1). The protein, which is encoded by the WP_011551853.1 gene, was identified as acidic LOX isoforms (Qian et al., 2017) and recombinant expression of the WP_011551854.1 gene also led to a catalytically active enzyme (An, Hong, & Oh, 2018). In addition, our database search identified two putative LOX genes in the genome of *M. fulvus* (WP_046712474.1 and SEU34910.1), which have not been characterized so far. To explore whether the WP_046712474.1 gene encodes for a functional LOX, we expressed the corresponding enzyme in different pro‐ and eukaryotic expression systems and characterized the recombinant protein with respect to its protein‐chemical and enzymatic properties. Our results indicate that *M. fulvus* expresses an arachidonic acid 12S‐lipoxygenating LOX isoform (MF‐LOX1), which only shares a low degree (20%) of amino acid identity with a recently characterized LOX isoform from *M. xanthus *(An et al., 2018) and with other pro‐ and eukaryotic LOX isoforms.

## 2 RESULTS

### 2.1 Database search and identification of putative LOX genes in the genome of* M. fulvus*

**Figure 1:**
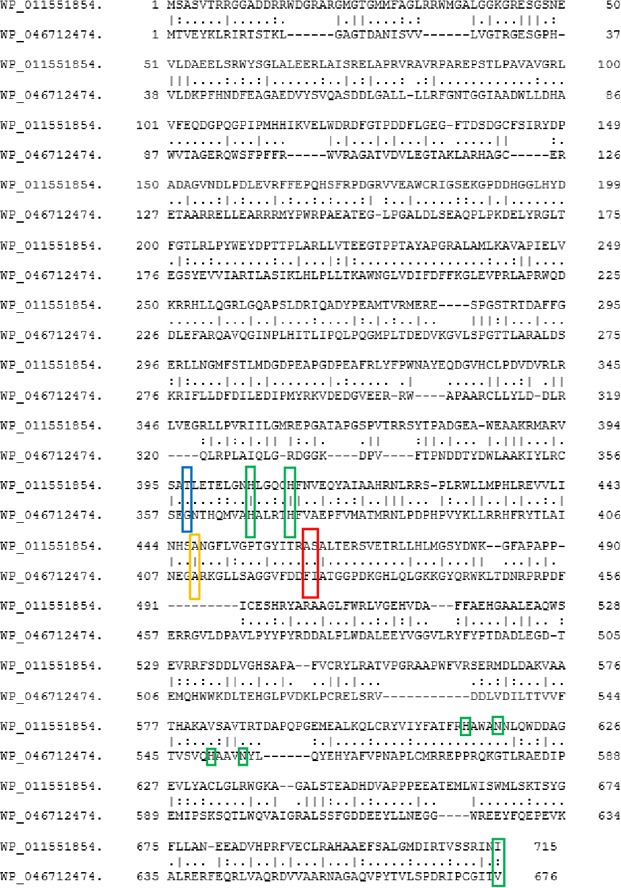
Dual amino acid alignment of *Myxococcus fulvus* LOX1 (MF‐LOX1, WP_046712474.1) and *M. xanthus *LOX2 (WP011551854.1). The putative iron ligands are framed in green, and the sequence determinants of the reaction specificity are color coded as follows: yellow—Coffa determinant, blue—Borngraber 1 determinant, red—Sloane determinants

Lipoxygenases (ALOX isoforms) rarely occur in prokaryotes but in *M. xanthus *two functional ALOX genes (WP_011551854.1 and WP_011551853.1) have recently been identified (An et al., 2018; Qian et al., 2017). In *Myxococcus fulvus (M. fulvus)*, which differs from *M. xanthus* with respect to structural and functional characteristics, no LOX isoforms have been described so far. When we searched the NCBI bacterial genome database for potential ALOX sequences, we detected two potential ALOX sequences (WP_046712474.1 and SEU34910.1) in *M. fulvus*. WP_046712474.1 encodes for a 676 amino acid protein (designated MF‐LOX1 in this paper), which involves two functional iron ligand clusters (C1 [His376 + His372] and C2 [His549 + Asn553]), which are essential for true ALOX sequences. The SEU34910.1 gene encodes for a 689 amino acid protein (designated MF‐LOX2 in this paper), which also involves two iron ligand clusters. The two proteins only share a low degree of amino acid identity (21.4%) and, thus, should exhibit different functionality. Amino acid alignments of these *M. fulvus* LOX (MF‐LOX1, MF‐LOX2) with the two *M. xanthus* enzymes (MX‐LOX1 and MX‐LOX2) indicated that MF‐LOX1 (WP_046712474.1) shares a low degree (19.7%) of amino acid identity with the well‐characterized MX‐LOX2 (WP_011551854.1). Although the iron clusters are conserved in the two sequences (Figure 1), the degree of overall similarity is limited. The degree of amino acid identity of MF‐LOX1 discovered here with the poorly characterized *M. xanthus* LOX (MX‐LOX1) is 86%. A similar degree of amino acid identity (85%) is shared between different mammalian ALOX15 orthologs (men vs. mouse, mouse vs. rats, rats vs. men, men vs. pigs etc.). Thus, it might be speculated that MF‐LOX1 (WP_046712474.1) and MX‐LOX1 (WP_011551854.1) also represent enzyme orthologs in two different *Myxococcus* species.

### 2.2 Recombinant expression of MF‐LOX1 in* E. coli*

**Figure 2:**
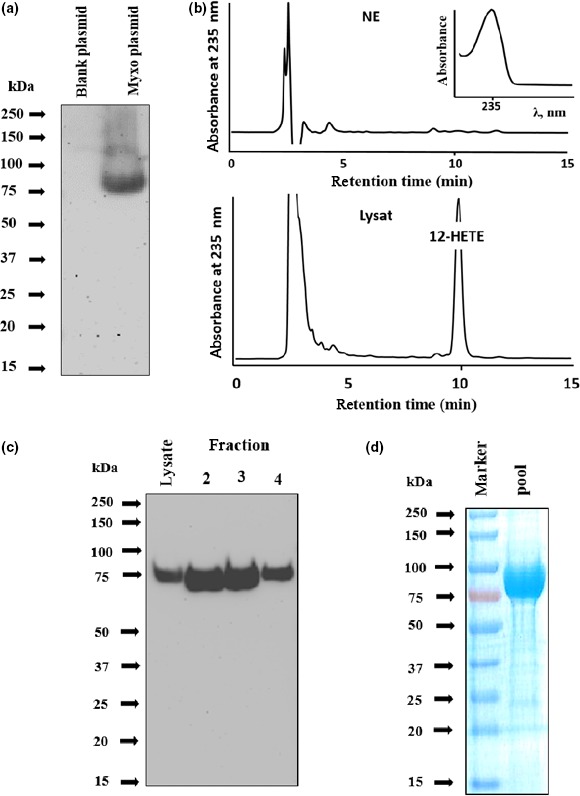
Bacterial expression and purification of recombinant *Myxococcus* *fulvus* LOX1 (MF‐LOX1). MF‐LOX1 was expressed as N‐terminal hexa‐his‐tag fusion protein in *Escherichia coli*. (a) Immunoblotting: Bacteria were lyzed by sonication and cell debris was removed. Aliquots of the bacterial lysis supernatant were applied to SDS‐PAGE, the separated proteins were transferred to a nitrocellulose membrane, and the membrane was probed with an anti‐his‐tag antibody. Left lane: *E. coli* transformed with empty plasmid. Right lane: *E. coli* transformed with recombinant plasmid. (b) LOX‐activity assay (RP‐HPLC): In vitro activity assays were carried out as described in Experimental Procedures, and the reaction products were analyzed by RP‐HPLC. Retention times of authentic standards are indicated. A nonenzyme (NE) incubation (PBS instead of the bacterial lysate supernatant) was carried out as negative control. (c) Protein purification: Affinity chromatography of the recombinant hexa‐his‐tag fusion protein on Ni‐agarose was carried out and aliquots of the different elution fractions were analyzed by immunoblotting. (d) Degree of purity of the final enzyme preparation: MF‐LOX1 expressed in bacterial cells was purified by affinity chromatography. The active fractions 2, 3, and 4 were pooled as shown in panel c, desalted and an aliquot was applied to SDS‐PAGE

To explore whether the MF‐LOX1 gene (WP_046712474.1) encode for a functional ALOX isoform, we expressed the corresponding enzyme as N‐terminal his‐tag fusion protein in *Escherichia coli*, purified the recombinant protein by affinity chromatography on Ni‐agarose, and characterized it with respect to its protein‐chemical and enzymatic properties. When *E. coli* cells were transformed with the recombinant expression plasmid, they express a his‐tag fusion protein, which migrates in SDS‐PAGE in the molecular weight range of 80 kDa (Figure 2a). No immunoreactive protein was observed when bacteria were transformed with an empty plasmid. To confirm the expression of a functional enzyme ALOX, activity assays were carried out (Figure 2b). The RP‐HPLC chromatograms and the UV spectrum of the major oxygenation product indicate the formation of a conjugated diene during the incubation period and this product comigrated with an authentic standard of 12‐HETE (lower trace). This compound was not detected in control incubations (no enzyme). Since 12‐ and 8‐HETE are not well separated under our chromatographic conditions, additional SP‐HPLC was carried out to resolve the two product isomers. Here, the major oxygenation product comigrated with 12‐HETE (data not shown). For more comprehensive characterization, we purified the recombinant enzyme by affinity chromatography on a Ni‐agarose column. As indicated in Figure 2c, the his‐tag fusion protein was bound at the affinity matrix and was recovered by eluting the column with increasing amounts of imidazole. The bulk of the recombinant protein was eluted in fractions 2, 3, and 4. To quantify the degree of purity of the final enzyme preparation, Coomassie staining of an SDS‐PAGE was carried out and densitometric evaluation suggested that the enzyme was >95% pure (Figure 2d). The expression yield, the degree of purity, and the catalytic activities of the final enzyme preparations are summarized in Table 1.

**Table 1:** Expression and purification efficiency of *Myxococcus fulvus *LOX1 (MF‐LOX1)

Parameter	*Escherichia* *coli* (*n* = 3)	Sf9 cells (*n* = 1)
Yield (mg/L culture fluid)	118.2 ± 17.3	92
Degree of purity (%)	>95	>95
Specific activity (s^−^^1^)	(3.1 ± 1.2) x 10^−^^2^	2.6 x 10^−^^2^

Note

MF‐LOX1 was expressed as N‐terminal hexa‐his‐tag fusion protein in *E. coli* and in Sf9 cells as described in Experimental Procedures. After affinity chromatography on Ni‐agarose, the catalytically active fractions were pooled and the readout parameters given in the table were determined (with EPA as substrate)

### 2.3 Recombinant expression of MF‐LOX1 in a eukaryotic expression system

**Figure 3:**
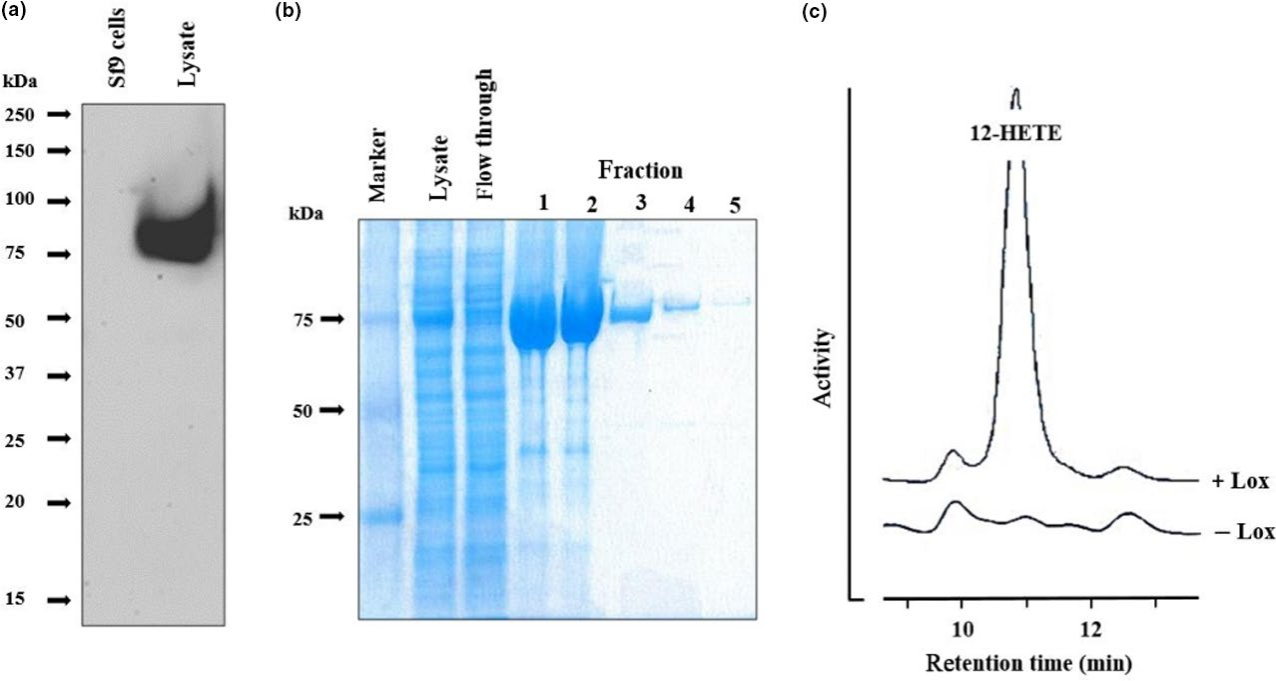
Eukaryotic expression of recombinant *Myxococcus fulvus* LOX1 (MF‐LOX1). MF‐LOX1 was expressed as N‐terminal hexa‐his‐tag fusion protein in Sf9 cells as described in Experimental Procedures. (a) Immunoblotting: Sf9 cells infected with the recombinant baculovirus were lyzed by repeated sonication and cell debris was removed by centrifugation. Aliquots of the cell lysis supernatant were applied to SDS‐PAGE, separated proteins were transferred to a nitrocellulose membrane, and the membrane was probed with an anti‐his‐tag antibody. Left lane: Lysis supernatant of uninfected Sf9 cells. Right lane: Lysis supernatant of Sf9 cells infected with recombinant baculovirus. (b) Affinity chromatography of MF‐LOX1 expressed in Sf9 cells: MF‐LOX1 was expressed in Sf9 cells and was purified by affinity chromatography on a Ni‐agarose column as described in Experimental Procedures. Aliquots of the different elution fractions were analyzed by SDS‐PAGE and stained with Coomassie blue. (c) LOX‐activity assay (RP‐HPLC): In vitro activity assays were carried out as described in Experimental Procedures using aliquots of the Sf9 cell lysate supernatant as enzyme source. The reaction products were analyzed by RP‐HPLC and a nonenzyme incubation (‐LOX, PBS instead of Sf9 cell lysate supernatant) was carried out as negative control

Evaluation of our activity assays suggested that the specific activity of the expressed MF‐LOX1 was considerably lower than that of *P. aeruginosa* (PA‐LOX) enzyme used in control incubations. To test whether the enzyme is more efficiently expressed in eukaryotic overexpression systems, we cloned the coding sequence into the pFastBac HT‐B expression vector and expressed the enzyme in Sf9 cells. Western blot analyses indicated that the recombinant enzyme is expressed (Figure 3a) and activity assays (Figure 3c) confirmed this conclusion. Here again, conjugated dienes that cochromatographed in RP‐HPLC with an authentic standard of 12S‐HETE were formed during the incubation period but these products were absent in control incubations. The enzyme could also be purified by affinity chromatography on Ni‐agarose (Figure 3b) and the final enzyme preparations exhibited a high (>95%) degree of electrophoretic homogeneity. The expression yield, the degree of purity, and the catalytic activities of the final enzyme preparations are summarized in Table 1.

The specific activity of the MF‐LOX1 preparation from SF9 cells was also low and comparison with the enzyme expressed in *E. coli* did not reveal major differences. For improvement, we attempted to overexpress the protein in HEK293 cells employing human ALOX15 as positive control. Although human ALOX15 was well expressed, comparative activity assays did not reveal any evidence for expression of MF‐LOX1. In fact, using the lysis supernatant of transfected HEK293 cells as enzyme source, we observed dominant formation of 15‐HETE for the human enzyme. In contrast, no 12‐HETE formation was observed when the lysis supernatant of MF‐LOX1 transfected HEK293 cells was employed (data not shown).

### 2.4 Protein‐chemical characterization of MF‐LOX1

**Figure 4:**
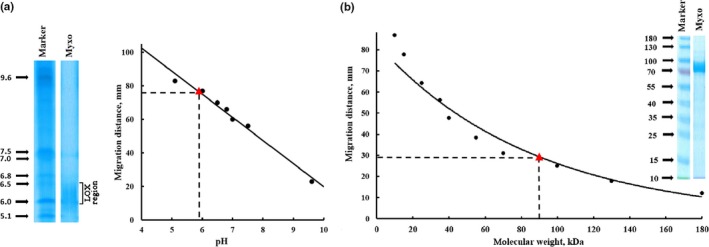
Protein‐chemical characteristics of MF‐LOX1. MF‐LOX1 was expressed as N‐terminal hexa‐his‐tag fusion protein in *Escherichia coli* as described in Experimental Procedures. (a) Native isoelectric point: An aliquot of the pooled Ni‐agarose fractions was applied to isoelectric focusing together with IP standards and from the relative migration distances the native IP of recombinant enzyme preparation was determined as 5.9. This value is in fair agreement with the theoretical IP calculated on the basis of the amino acid composition (5.8). (b) Apparent molecular weight: An aliquot of the pooled Ni‐agarose fractions was applied to SDS‐PAGE together with molecular weight standards and from the relative migration distances the experimental molecular weight of the recombinant enzyme preparation was determined as 90 kDa. This value is in fair agreement with the theoretical molecular weight calculated on the basis of the amino acid composition (80 kDa)

The theoretical molecular weight calculated from the amino acid sequence of our MF‐LOX1 construct including the hexa‐his tag and the linker peptide was 79.800 Da. From SDS‐PAGE of the purified enzyme, a molecular weight of 90.3 kDa was concluded (Figure 4b). Isoelectric focusing (Figure 4a) indicated several protein bands migrating in the pH region between 5.1 and 6.5 and these data suggest a structural microheterogeneity of our final enzyme preparation. Nevertheless, an IP in the pH region between 6.1 and 6.5 is consistent with the theoretical IP of 5.8, which was deduced from the amino acid composition. Because of technical reasons, the N‐terminal amino acid sequence of the recombinant protein was elongated by 32 amino acids when compared with the native protein. The final sequence reads: Met‐Gly‐Ser‐Ser‐*HIS‐HIS‐HIS‐HIS‐HIS‐HIS*‐Ser‐Ser‐Gly‐Leu‐Val‐Pro‐Arg‐Gly‐Ser‐His‐Met‐Ala‐Ser‐Met‐Tre‐Gly‐Ala‐Asn‐Gly‐Ser‐Gly‐Ser‐**Met**‐Thr. The bolded Met constitutes the starting methionine of the native protein and the italized capital letters represent the hexa‐his tag. The length of the additional N‐terminal peptide (32 additional amino acids), which includes the hexa‐his‐tag sequence, is mainly related to our cloning strategy and to the localization of the HindIII recognition sequence within the multicloning site of the expression vector.

All LOX isoforms characterized so far involve either iron or manganese as catalytically active transition metal (Ivanov et al., 2010; Wennman, Karkehabadi, & Oliw, 2014). To explore whether MF‐LOX1 carries manganese or iron as catalytically active constituent, we quantified the content of these transition metals in our purified enzyme preparation by atomic absorption spectroscopy. For this purpose, the enzyme preparation was desalted and aliquots of the desalting buffer were used for control measurements. We found that the manganese content of the enzyme preparation was lower than in the desalting buffer and these data suggest that MF‐LOX1 does not involve manganese. Quantification of the iron content yielded significantly higher iron levels than in the desalting buffer (Table 2), but calculation of the iron load suggested that only 5.6% of wild‐type MF‐LOX1 expressed in *E. coli* carried an iron ion. Unfortunately, all attempts to improve the iron load (iron supplementation of the fermentation sample, mutagenesis studies of the iron ligands, in vitro iron incorporation into the purified protein after incorporation experiments) were not successful. To exclude the possibility that MF‐LOX1 involves other transition metals as catalytically essential constituent, we next determined the copper and zinc concentrations in our enzyme preparation. However, both transition metals were below the detection limits. When we quantified the iron content of the Phe424Ile+Ile425Met double mutant, we observed a fourfold higher iron load. This observation is quite interesting since this double mutant exhibited a 4.7‐fold higher specific activity (Table 4). Thus, the major reason for the low catalytic activity of recombinant MF‐LOX1 (Table 1) is its low iron load. The mechanistic basis for the unusually low iron load of MF‐LOX1 remains unclear but possible scenarios are discussed later on in the manuscript.

**Table 2:** Iron content of pure recombinant MF‐LOX1 and its mutant

Parameter	Iron concentration (µmol/L)	Protein concentration (µmol/L)	Iron load (%)
Wild‐type	0.750	13.44	5.6
SL (Phe424lle + Ile425Met)	0.835	3.838	22

Note

MF‐LOX1 and Phe424Ile + Ile425Met mutant were expressed as N‐terminal hexa‐his‐tag fusion protein in *Escherichia* *coli* as described in Experimental Procedures. Aliquots of the pooled Ni‐agarose fractions (Figure 2d) were used for quantification of the iron content

### 2.5 Enzymatic characterization of MF‐LOX1

**Figure 5:**
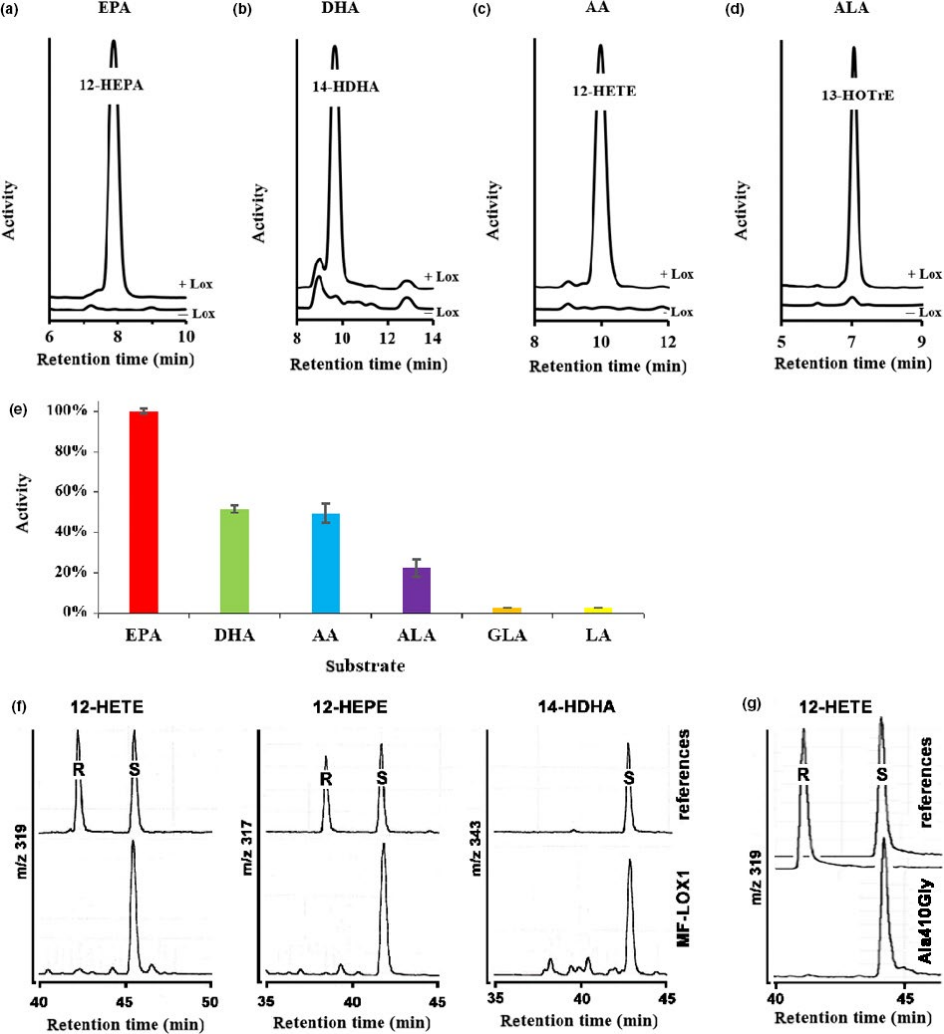
Substrate specificity of the MF‐LOX1 and enantioselectivity of product formation. MF‐LOX1 was expressed as N‐terminal hexa‐his‐tag fusion protein in *Escherichia coli*. Aliquots of the pooled Ni‐agarose fractions (Figure 1c) were employed for activity assays (see Experimental Procedures) with different fatty acids as substrates. The reaction products were analyzed by RP‐HPLC recording the absorbance at 235 nm. Authentic standards of 12‐HEPE, 14‐HDHA, and 12‐HETE were prepared using recombinant human ALOX12 (Kutzner et al., 2017). (a) Product pattern of EPA oxygenation, (b) product pattern of DHA oxygenation, (c) product pattern of AA oxygenation, (d) product pattern of ALA oxygenation, (e) substrate specificity: The conjugated dienes formed during the incubation period were quantified and the product formation from EPA was set 100%. (f) Analysis of the enantiomer composition of the major oxygenation products formed by MF‐LOX1 from C20 and C22‐polyenoic fatty acids. The enantiomer composition was determined by chiral phase LC‐MS (see Experimental Procedures). (g) Analysis of 12‐HETE enantiomer composition formed by the Ala410Gly mutant

To explore the substrate specificity of recombinant MF‐LOX1, we tested several omega‐6 (linoleic acid, gamma‐linolenic acid, arachidonic acid) and omega‐3 (alpha‐linolenic acid, eicosapentaenoic acid, docosahexaenoic acid) polyenoic fatty acids as substrate. Here, we found that MF‐LOX1 most effectively oxygenated eicosapentaenoic acid (Figure 5e). Docosahexaenoic acid, arachidonic acid, and alpha‐linolenic were also well oxygenated. In contrast, no oxygenation products were detected when gamma‐linolenic acid and linoleic acid were used as substrate. To identify the chemical structure of the reaction products, we compared in RP‐HPLC the reaction products formed by MF‐LOX1 with those of human recombinant ALOX15. Here, we found that the major oxygenation product of EPA oxygenation (Figure 5a) comigrated with the minor oxygenation product formed from this fatty acid by the human enzyme, which is 12‐HEPA (Kutzner et al., 2017). Similarly, the major oxygenation product formed from DHA and AA by MF‐LOX1 (Figure 5b,c) cochromatographed in RP‐HPLC with the minor oxygenation products formed from these substrates (14‐HDHA, 12‐HETE) by the human enzyme (Kutzner et al., 2017). ALA was oxygenated by MF‐LOX1 (Figure 5d) and human ALOX15 to an identical major oxygenation product (13‐HOTrE).

To define the degree of optical purity of the major oxygenation products, we carried out chiral phase LC‐MS and the corresponding chromatograms are shown in Figure 5f. For this purpose, the chromatograms were followed at m/z 319 (12‐HETE), 317 (12‐HEPE), and 343 (14‐HDHA). It can be seen that for all of these major oxygenation products, the S‐isomer prevails and that the corresponding R‐enantiomers are only formed in small amounts. Exact quantification of the S/R ratio is given in Table 3. Taken together, these data indicate a high degree of stereo‐chemical control of the oxygenase reaction.

**Table 3:** Enantiomer composition of major conjugated dienes formed from EPA (12‐HEPE), DHA (14‐HDHA), and AA (12‐HETE)

Metabolite	*S *(%)	*R *(%)
12‐HEPE	97.5	2.5
14‐HDHA	95	5
12‐HETE	95	5

Note

MF‐LOX1 was expressed as N‐terminal hexa‐his‐tag fusion protein in *Escherichia* *coli* as described in Experimental Procedures. Aliquots of the pooled Ni‐agarose fractions (Figure 2d) were used for activity assays. The major conjugated dienes formed were isolated by RP‐HPLC and separation of the enantiomers was carried out by chiral phase LC/MS (see Experimental Procedures)

**Figure 6:**
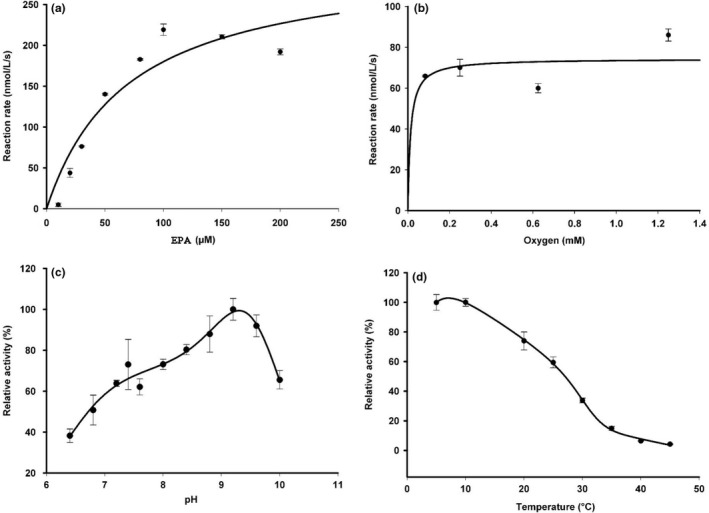
Kinetic properties of MF‐LOX1. MF‐LOX1 was expressed as N‐terminal hexa‐his‐tag fusion protein in *Escherichia coli*. Aliquots of the pooled Ni‐agarose fractions (Figure 2d) were used to determine basic kinetic properties of the enzyme. (a) Fatty acid substrate affinity: Aliquots of the enzyme preparation were incubated for 1 min with different concentrations of EPA and the amounts of conjugated dienes formed (RP‐HPLC) were used to construct Michaelis–Menten diagram. (b) Oxygen affinity: Different volumes of anaerobic (argon flushed) PBS were mixed with different volumes of oxygen saturated (oxygen flushed) PBS. After addition of a partially anaerobized methanolic EPA solution (160 µM final concentration), the reaction was started by the addition of 50 µl partially anaerobized enzyme solution. After a 1‐min incubation period, the formed conjugated dienes were quantified by RP‐HPLC. The experimental raw data were fitted to the Michaelis–Menten equation to extract the Km‐value. (c) pH dependence: The reaction buffer was prepared by mixing equal volumes of 10 mM phosphate and 10 mM borate solutions adjusting the different pH values by the addition of different volumes of 1 M NaOH or 1 M HCl. The amounts of conjugated dienes formed during a 1‐min incubation period (RP‐HPLC) were quantified to establish the pH profile. (d) Temperature dependence: Activity assays (see Experimental Procedures) were carried out at different reaction temperatures and the amounts of conjugated dienes formed (RP‐HPLC) were quantified as readout parameter

To obtain more detailed information on the reaction kinetic of MF‐LOX1, we quantified its substrate affinity using eicosapentaenoic acid as model substrate. From Figure 6a, it can be seen that the enzyme follows Michaelis–Menten kinetics and a Km‐value of about 70 µM was determined. This value is in the same range (50 µM) as determined for the arachidonic acid oxygenase activity of *P. aeruginosa* LOX (Banthiya et al., 2016) under comparable experimental conditions (lack of any detergents). However, more detailed inspection of the Michaelis–Menten diagram (Figure 6a) suggested substrate inhibition of the enzyme at higher substrate concentrations (150, 200 µM). When we excluded the activity data measured at these substrate concentrations from our kinetic modeling, we obtained a Km‐value of 427 µM. This constant was considerably higher than the corresponding value obtained when these reaction rates were included in Km determination. Under *V*_max_ conditions (eicosapentaenoic acid as substrate), a molecular turnover rate of (3.1 ± 1.2) x 10^−2^/s was determined. This value is more than two orders of magnitude lower than the turnover rate determined for linoleic acid oxygenation by human ALOX15 (Ivanov, Kuhn, & Heydeck, 2015) and four orders of magnitude lower than that of the PA‐LOX (Banthiya et al., 2016). The molecular basis for the low specific activity of MF‐LOX1 remains unclear but the low iron load of the recombinant enzyme may contribute.

For several LOX isoforms, it has been shown that oxygen enters the active site *via* preformed or dynamic oxygen channels (Knapp, Seebeck, & Klinman, 2001; Saam, Ivanov, Walther, Holzhutter, & Kuhn, 2007; Xu, Mueser, Marnett, & Funk, 2012). The oxygen affinities of different LOX isoforms are rather high and oxygen Km values in the lower micromolar range have been reported (Egmond, Brunori, & Fasella, 1976; Juranek, Suzuki, & Yamamoto, 1999; Knapp & Klinman, 2003; Ludwig et al., 1987). On the other hand, wild‐type *P. aeruginosa* LOX has a Km for oxygen of about 0.4 mM indicating that under normoxic conditions this enzyme does not work at substrate saturation (Kalms et al., 2017). To estimate the oxygen affinity of MF‐LOX1, we performed activity assays at different oxygen concentrations and quantified the amounts of conjugated dienes formed during a 1‐min oxygenation period. When we fitted the obtained activity data to the Michaelis–Menten equation (Figure 6b), a low Km for oxygen (12 µM) was concluded. It should be stressed at this point that in quantitative terms this value is not very precise because of two reasons: (a) Most measurements were carried out in the oxygen concentration range close to oxygen saturation. (b) It is impossible completely exclude oxygen by flushing the reaction buffer with argon gas, and thus, the actual oxygen concentrations in the incubation samples are rather rough estimates. Nevertheless, our experimental data clearly indicate that the catalytic activity of MF‐LOX1 could not be improved by increasing the oxygen concentrations. In other words, MF‐LOX1 exhibits a high oxygen affinity and this conclusion is consistent with experimental data obtained for different mammalian ALOX isoforms (Juranek et al., 1999).

LOXs exhibit different pH profiles but most isoforms have neutral or alkaline pH optima. Recently, an acidic LOX isoforms was identified in *M. xanthus* (WP_011551853.1), which showed a pH optimum of 3 (Qian et al., 2017). This enzyme shares a high degree (86%) of amino acid identity MF‐LOX1 but it prefers linoleic acid over arachidonic acid. When we recorded the pH profile MF‐LOX1 (Figure 6c), we observed the pH optimum at 9.5.

Finally, we determined the temperature dependence of EPA oxygenation by MF‐LOX1 (Figure 6d). Here, we observed similar catalytic activities in the temperature range between 5°C and 15°C. When we increased the reaction temperatures above 15°C, a steady decline of the catalytic activity was observed. The molecular basis for this unusual temperature dependence has not been explored but it may be related to a limited thermostability of the enzyme.

### 2.6 Mutagenesis of catalytically important amino acids

For eukaryotic ALOX isoforms, several hypotheses explaining the mechanistic basis of their reaction specificities have been developed. The Triad Concept (Borngraber et al., 1999; Ivanov et al., 2015; Vogel et al., 2010), which was developed for mammalian ALOX15 orthologs, suggests that three regions of the primary structure of these enzymes (triad determinants) are important for their reaction specificities. To identify the triad determinants of MF‐LOX1, we carried out multiple sequence alignments of this enzyme with different mammalian ALOX15 orthologs and found that Phe353 of human and rabbit ALOX15 (Borngraber‐1 determinant) aligned with Gly359 of MF‐LOX1. Similarly, Phe424 and Ile425 of MF‐LOX aligned with Ile418 and Met419 of human and rabbit ALOX15 (Slone determinant). Finally, Ile593 of mammalian ALOX15 orthologs (Borngraber‐2 determinant) aligned with Ile603 of MF‐LOX1. Next, we mutated the two most important triad determinants (Borngraber‐1 and Sloane determinants) of MF‐LOX1. For this purpose, we first mutated Gly359 introducing a more bulky Phe, which is present at this position in human and rabbit ALOX15. Unfortunately, the Gly359Phe mutant was catalytically inactive (Table 4), and thus, no functional conclusions could be drawn. When we applied a similar mutagenesis strategy to the Sloane determinants of MF‐LOX1 (creation of the Phe424Ile+Ile425Met double mutant), we obtained an enzyme species, which exhibited an almost fivefold higher catalytic activity than the wild‐type enzyme. However, the reaction specificity of this gain‐of‐function mutant was identical to that of the wild‐type enzyme (Table 4). These data suggest that the Triad Concept (Ivanov et al., 2015) might not be applicable for MF‐LOX1.

**Table 4:** Mutagenesis of the putative sequence determinants of *Myxococcus fulvus* LOX1 (MF‐LOX1)

Enzyme	Relative activity (%)	12‐HETE (%)	15‐HETE (%)
Wild‐type	100	95	5
BG1 (Gly359Phe)	<5	—	—
SL (Phe424Ile + Ile425Met)	475	95	5
CO (Ala410Gly)	109	>97.5	<2.5

Notes

BG1: Borngraber 1 determinant; CO: Coffa/Brash determinant; SL: Sloane determinants.

MF‐LOX1 was expressed as N‐terminal hexa‐his‐tag fusion protein in *Escherichia* *coli* and purified by affinity chromatography on Ni‐agarose as described in Experimental Procedures. The sequence determinants of MF‐LOX1 were identified by amino acid sequence alignment (Figure 1). Aliquots of the pooled Ni‐agarose fractions (Figure 2d) were used for activity assays and the reaction products of arachidonic acid oxygenation were quantified by RP‐HPLC (see Experimental Procedures).

Previous multiple sequence alignments have indicated that most S‐LOXs carry an Ala at a critical position of their primary structures. In contrast, most R‐LOXs involve a smaller Gly at this position and Ala‐to‐Gly exchange increase the relative share of R‐lipoxygenation products (Coffa & Brash, 2004; Coffa, Schneider, & Brash, 2005; Vogel et al., 2010). Although this concept was not applicable for zebrafish LOX1 (Haas et al., 2011; Jansen et al., 2011), we tested the impact of Ala410Gly exchange on MF‐LOX1 and found that this mutation did neither alter the catalytic activity nor the positional specificity of the enzyme. When we analyzed by chiral phase HPLC the enantiomer composition of the major reaction product formed by the Ala410Gly mutant, we detected dominant 12S‐HETE formation. Thus, the enantioselectivity was neither altered by this mutation (Figure 5g). Taken together, our mutagenesis data suggest that MF‐LOX1 does neither follow the Triad Concept nor the Ala‐vs.‐Gly Hypothesis.

### 2.7 Biomembrane oxygenase activity

Some ALOX isoforms are capable of oxygenating membrane phospholipids, which leads to disruption of membrane integrity (Aldrovandi et al., 2018; Kühn et al., 1993; Pekarova, Kuhn, Bezakova, Ufer, & Heydeck, 2015; Schewe, Halangk, Hiebsch, & Rapoport, 1975; Takahashi et al., 1993). To test whether MF‐LOX1 also exhibits biomembrane oxygenase activity, we incubated beef heart submitochondrial particles as model membranes with purified native rabbit ALOX15 and recombinant MF‐LOX1 for 15 min. After the incubation period, the reaction products were reduced, lipids were extracted, hydrolyzed and the resulting free fatty acids (sum of HODE + HETE isomers, linoleic acid [LA] + arachidonic acid [AA]) were analyzed by RP‐HPLC. To quantify the degree of membrane phospholipid oxygenation, we calculated the molar HODE + HETE/LA + AA ratio (Aldrovandi et al., 2018; Kuhn, Belkner, Wiesner, & Brash, 1990). In Table 5, it can be seen that under our in vitro conditions the membrane oxygenase activity of rabbit ALOX15 was threefold higher than that of MF‐LOX1. This difference was even more pronounced when we normalized the membrane oxygenase activity to the amounts of enzyme added as catalysts. For MF‐LOX, we applied 7 mg/ml of purified enzyme but for rabbit ALOX15 7 µg/ml. It should, however, been stressed that the specific fatty acid oxygenase activity of the rabbit ALOX15 is orders of magnitude higher than that of MF‐LOX1 (Table 1).

**Table 5:** MF‐LOX1 does not exhibit membrane oxygenase activity

Enzyme	OH‐PUFA/PUFA ratio (mole %)
Rabbit ALOX15	0.65 ± 0.28 (*n* = 2)
MF‐LOX1	0.22 ± 0.03 (*n* = 4)

Note

Purified recombinant MF‐LOX1 (7 mg/ml) and pure native rabbit ALOX15 (7 µg/ml) were incubated in PBS with beef heart submitochondrial membranes (1.2 mg/ml) for 15 min at room temperature. After the incubation period, the reaction products were reduced with NaBH4 and the pH was adjusted to 3.5 with acetic acid. Total lipids were extracted (Bligh & Dyer, 1959), ester lipids were hydrolyzed, and the resulting free fatty acids (sum of HODE + HETE isomers, linoleic acid [LA] + arachidonic acid [AA]) were analyzed by RP‐HPLC. To quantify the degree of membrane phospholipid oxygenation, we calculated the molar HODE + HETE/LA + AA ratio (OH‐PUFA/PUFA ratio) and the data obtained for the noenzyme control were subtracted.

## 3 DISCUSSION

### 3.1 Occurrence of LOX isoforms in different myxobacterial species

In bacteria, LOXs rarely occur (Horn et al., 2015) but functional bacterial LOXs have been identified in different cyanobacteria (Andreou, Göbel, Hamberg, & Feussner, 2010b; Gao, Boeglin, & Brash, 2010; Kim, An, Lee, & Oh, 2015; Zheng, Boeglin, Schneider, & Brash, 2008) and proteobacteria (Garreta et al., 2013; Kim et al., 2015; Vance et al., 2004). More recently, in the genome of *M. xanthus*, LOX genes have been identified (WP_011551853.1, WP_011551854.1, ABF 88826.1) and two of them have been shown to encode for distinct functional LOX isoforms, which only share a minor degree of amino acid conservation (Table 6) and exhibit distinct catalytic activities (An et al., 2018; Qian et al., 2017). In *M. fulvus,* which constitutes a different species within the bacterial genus *Myxococcus*, no LOX genes have been described so far. We recently screened the genome of *M. fulvus *for potential LOX genes and identified two potential LOX genes (WP_046712474.1 and SEU34910.1). The corresponding proteins share a low degree of amino acid identity (Table 6), and thus, they are apparently not closely related. In fact, human ALOX15 and human ALOX5, which exhibit different biological functions, share a higher degree of amino acid identity (40%). When we compared the amino acid identity scores of the two putative *M. fulvus* LOX with the two *M. xanthus* enzymes (Table 6), we found that the enzyme characterized in this study (WP_046712474.1, MF‐LOX1) only shares a low degree (19.7%) of amino acid identity with the well‐characterized 12S‐LOX of *M. xanthus* (An et al., 2018). In contrast, the enzyme appears to be more closely related to the poorly characterized *M. xanthus* LOX (Qian et al., 2017) since the amino acid identity score was close to 86% (Table 6). Similar identity scores were found when mouse (74%), rat (75%), and pig (86%) ALOX15 orthologs were compared with the human enzyme. Thus, it might well be that the *M. fulvus* LOX characterized here (WP_046712474.1, MF‐LOX1) may constitute the functional equivalent of MX‐LOX1 (WP_011551853.1), which has not been characterized very well (Qian et al., 2017). However, the currently available functional data do not provide major evidence for such a close functional relation: (a) MX‐LOX1 has been described as acidic LOX with a pH optimum of 3.0 (Qian et al., 2017). In contrast, we detected an alkaline pH optimum (pH_opt_ of 9.5) for MF‐LOX1 (Figure 6c). (b) MX‐LOX1 prefers linoleic acid over arachidonic acid (Qian et al., 2017). In contrast, we found that linoleic acid is a poor substrate for MF‐LOX1 when compared with arachidonic acid, eicosapentaenoic acid, and docosahexaenoic acid (Figure 5e). (c) MX‐LOX1 is rather stable and exhibits a temperature optimum of 30°C (Qian et al., 2017). In contrast, MF‐LOX1 is unstable and exhibits an unusual temperature dependence with a T_opt_ at 10°C. Although these kinetic parameters suggest that MX‐LOX1 and MF‐LOX1 may not be closely related, they still might constitute functional equivalents in the two different *Myxococcus* species. More detailed functional characterization of MX‐LOX1 is needed to draw more definite conclusions.

**Table 6:** Degree of amino acid conservation of myxobacterial LOX isoforms

Enzyme	WP_011551853.1 (MX‐LOX1)	WP_011551854.1 (MX‐LOX2)	WP_046712474.1 (MF‐LOX1)	SEU34910‐1 (MF‐LOX2)
WP_011551853.1 (MX‐LOX1)	100	19.7	85.8	21.1
WP_011551854.1 (MX‐LOX2)	19.7	100	19.7	85.7
WP_046712474.1 (MF‐LOX1)	85.8	19.7	100	21.4
SEU34910–1 (MF‐LOX2)	21.1	85.7	21.4	100

Note

For the time being, two different LOX isoforms (An et al., 2018; Qian et al., 2017) have been described in *Myxococcus* *xanthus* (MX‐LOX1—WP_011551853.1, MX‐LOX2—WP_011551854.1). When we screened the currently available bacterial genomes for LOX‐like sequences, we detected two potential LOX genes in *M. fulvus.* We carried out dual amino acid alignments and observed variable degrees of amino acid conservation between the different myxobacterial LOX isoforms

### 3.2 Low iron content and limited catalytic activity

Transition metal analyses of our final enzyme preparation indicated that recombinant MF‐LOX1 does neither involve manganese nor copper and zinc as catalytically active transition metal. Unfortunately, we also found that the iron content was rather low so that an iron load of only 5.6% was calculated for the wild‐type enzyme. This low iron saturation might be discussed as molecular basis for the low catalytic turnover rate ([3.1 ± 1.2] x 10^−2^ s^−1^). If one calculates the putative catalytic activity for an enzyme preparation with 100% iron load, a molecular turnover rate of about 0.55 ± 0.21 s^−1^ results. This value is still lower than the turnover rates determined for rabbit and human ALOX15 (Ivanov et al., 2015; Kühn et al., 1993), soybean LOX1 (Egmond et al., 1976; Maccarrone et al., 2001), and *P. aeruginosa* LOX (Banthiya et al., 2016; Garreta et al., 2013). The molecular basis for the low iron affinity of MF‐LOX1 has not been explored in detail. However, iron supplementation of the fermentation sample, which was successful for the *Cyanothece *sp. LOX (Andreou, Gobel, Hamberg, & Feussner, 2010a), did neither improve the iron load nor the catalytic activity of MF‐LOX1. Moreover, parallel expression of *P. aeruginosa* LOX led to a recombinant protein exhibiting an iron load of 100% (Banthiya et al., 2016). Since the expression levels of the two proteins were comparable, one can exclude that problems with the iron incorporating machinery are major reasons for the low efficiency of iron incorporation for MF‐LOX1. These data rather suggest that MF‐LOX1 protein has a low iron affinity and this property might be related to its 3D‐structure. It might be possible that regular folding of the polypeptide chain of MF‐LOX1 requires special folding catalysts, which are present in *M. fulvus* but not in *E. coli* and Sf9 cells. We are currently attempting to crystallize wild‐type MF‐LOX1 and its Phe424Ile + Ile425Met double mutant to obtain direct structural evidence for this hypothesis. Discussing the structural basis for the low iron affinity of MF‐LOX1, it may also be of interest that the C‐terminal iron‐binding cluster (**His**‐Ala‐Ala‐Val‐**Asn**; iron liganding amino acids are bolded) of this enzyme does not well align with the corresponding sequence of *M. xanthus* LOX2 (MX‐LOX2, Figure 1). However, since the sequence similarities of the two enzymes in this particular region of the primary structure are not very pronounced, alignment artifacts might be possible. On the other hand, both iron ligand clusters of MF‐LOX1 align well with the corresponding amino acid of human and rabbit ALOX15, which makes alignment artifacts unlikely.

### 3.3 Biological activity of bacterial LOX

Mammalian ALOX isoforms have been implicated in cell differentiation but also in the pathogenesis of various diseases (Haeggstrom & Funk, 2011; Kuhn et al., 2015). Unfortunately, much less is known on the biological relevance of bacterial LOXs but several scenarios have been suggested: (a) Biofilm formation: When planktonic bacteria recognize specific attachment sites, when they suffer from malnutrition or when confronted with sublethal concentrations of antibiotics they form biofilms (Gupta, Sarkar, Das, Bhattacharjee, & Tribedi, 2016; Hoffman et al., 2005; Horn & Lackner, 2014; Karatan & Watnick, 2009). Recent cell culture experiments suggested that PA‐LOX is required for biofilm formation when *P. aeruginosa* (PA) interacts with host cells (Deschamps et al., 2016). The molecular basis for this phenomenon has not been studied in detail but the enzyme might be involved in intercellular lipid signaling. Similar communication mechanisms may exist for myxobacteria since these prokaryotes exhibit a pronounced social behavior (Velicer, Kroos, & Lenski, 1998; Welch & Kaiser, 2001). (b) Invasive growth and nutrient mobilization: Bacteria compete with other living individuals for nutrients and one way to get such nutrients is lysis of competitor cells. Unfortunately, potential target cells are protected by the plasma membranes. However, PA‐LOX is capable of oxidizing the membrane lipids of competitor cells, which leads to cellular lysis (Aldrovandi et al., 2018; Banthiya et al., 2016). Although the membrane oxygenase activity of MF‐LOX1 was limited in our in vitro experiments, the in vivo situation may be different. (c) Evasion strategies: When bacteria infect more complex organisms, the hosts’ immune system fights the pathogen. However, some pathogens developed evasion strategies to silence the immune response. LOXs have been implicated in the biosynthesis of anti‐inflammatory mediators, such as lipoxins, resolvins, and maresins (Ryan & Godson, 2010; Serhan & Chiang, 2013; Serhan, Dalli, Colas, Winkler, & Chiang, 2015), which downregulate the intensity of the inflammatory reaction. Recent in vitro experiments indicated that PA‐LOX exhibits lipoxin synthase activity (Banthiya et al., 2016). (d) Oxygen sensing: The oxygen affinity of most mammalian LOXs varies between 3 and 30 μM (Juranek et al., 1999). However, PA‐LOX (Kalms et al., 2017) exhibits a low oxygen affinity (Km > 400 μM). Since such kinetic properties are characteristic for sensor proteins (Berra et al., 2003), PA‐LOX might be involved in oxygen sensing. However, MF‐LOX1 exhibits a rather high oxygen affinity, and thus, its suitability to function as oxygen sensor is limited. (e) PUFA toxicity: Unsaturated fatty acids are toxic for many bacteria (Greenway & Dyke, 1979; Raychowdhury, Goswami, & Chakrabarti, 1985). Oleic acid is toxic for Streptococcus pyogenes M49 but this bacterium expresses a fatty acid double bond hydratase to metabolize this toxin (Volkov et al., 2010). Although recombinant PA‐LOX does not oxygenate oleic acid, it might contribute to detoxification of other unsaturated fatty acids.

## 4 EXPERIMENTAL PROCEDURES

### 4.1 Chemicals

The chemicals were obtained from the following sources: arachidonic acid, linoleic acid, alpha‐linolenic acid, gamma‐linolenic acid, eicosapentaenoic acid, and docosahexaenoic acid from Sigma (Taufkirchen, Germany); HPLC standards of 12(±)*‐*HETE, 12S‐HETE, 15(±)‐HETE, 15*S*‐HETE, 13*S*‐HODE, 13(±)‐HODE from Cayman Chem. (distributed by Biomol, Hamburg, Germany); sodium borohydride from Life Technologies, Inc. (Eggenstein, Germany); HPLC solvents from Baker (Deventer, The Netherlands); antibiotics and isopropyl‐β‐thiogalactopyranoside (IPTG) from Carl Roth GmbH (Karlsruhe, Germany); restriction enzymes from Thermo Fisher Scientific‐Fermentas (Schwerte, Germany); and the *E. coli *strain (Rosetta(DE3) pLysS) from Invitrogen (Carlsbad, USA). Oligonucleotide synthesis was performed at BioTez (Berlin, Germany). Nucleic acid sequencing was carried out at Eurofins MWG Operon (Ebersberg, Germany).

### 4.2 Bacterial expression and purification of MF‐LOX1

Genomic DNA of *M. fulvus* was purchased from the Leibnitz‐Institute DSMZ (German Collection of Microorganisms and Cell Culture; Klon DSM 16525), and the LOX cDNA sequence (NZ_FOIB01000010 REGION: complement 205294–207324, WP_074957772.1) was amplified using specifically designed primers (upstream: GGA TCC ATG ACT GTC GAG TAC AAG, downstream: AAG CTT TTA GAC GGT GAT GCC GCA). These primers involved the recognition sequences of the restriction enzymes *BamHI *(upstream primer) and *HindIII* (downstream primer) for convenient inclusion of the amplification product into the pro‐ and eukaryotic expression vectors. After amplification, the PCR product was purified (Nucleospin Gel and PCR Clean‐up, Macherey & Nagel, Düren, Germany) and cloned into a TOPO TA 2.1 vector (Thermo Fisher Scientific, Schwerte, Germany). *E. coli *XL‐1‐Blue cells were transformed with the recombinant plasmid, and the resulting clones were tested for the insert after plasmid preparation (GENEJET PL MINIPREP, Thermo Fisher Scientific, Schwerte, Germany) and digestion of the plasmid with *BamHI* and *HindIII. *A positive clone was selected and involvement of the insert was checked by sequencing (Eurofins Genomics, Ebersberg, Germany). From this LOX‐positive clone, a medium‐scale plasmid preparation was performed using the plasmid preparation kit Nucleobond Xtra Midi Plus from Macherey & Nagel. After preparative digestion of the recombinant plasmid with *BamHI* and *HindIII*, the digestion product was ligated (Rapid DNA Ligations‐kit, Thermo Fisher Scientific) into the expression vector pET28b (Thermo Fisher Scientific). Competent bacteria were transformed with the ligation mixture, plated, and then selected for antibiotic resistance. Two positive clones were picked and nucleotide sequencing confirmed proper insertion of the LOX insert (pET28‐MF‐LOX1). From one of these clones, plasmid DNA was prepared, the insert was sequenced, and this plasmid was further employed for the expression studies. In detail, bacterial expression involved the following steps: Competent *E. coli cells *(Rosetta2 DE3 pLysS or BL21 DE3) were transformed with 100 ng of the pET28b‐MF‐LOX1 plasmid and grown on kanamycin/chloramphenicol containing agar plates. Two 1 ml bacterial precultures (LB medium with 50 μg/ml kanamycin/35 µg/ml chloramphenicol) were inoculated and grown at 37°C for 6 hr and 180 rpm agitation. This pre‐culture was then checked for optical density (should have an OD_650_ of 0.1–0.15 at a dilution of 1:50) and added to a 50 ml main culture as recommended by the vendor (BioSilta, Berlin, Germany). The main culture was grown overnight at 30°C and the culture was continuously shaken at 250 rpm in Ultra Yield flasks (BioSilta Ltd., St. Ives, Great Britain). Expression of the recombinant enzyme was induced by adding 1 mM (final concentration) IPTG to the main culture and afterward the culture was incubated over night at 22°C and 250 rpm agitation. Bacteria were harvested by centrifugation and the resulting pellet was reconstituted in 5 ml PBS containing 2 mM EDTA. Bacteria were lyzed by sonication (Digital Sonifier, W‐250D Microtip Max 70% Amp, Model 102C (CE); Branson Ultraschall, Fürth, Germany), cell debris was removed by centrifugation (10 min, 15,000 *g*, 4°C), and the lysate supernatant was employed for further enzyme purification. To remove foreign bacterial proteins from the lysis supernatant, we carried out affinity chromatography on a Ni‐NTA‐Agarose column. For this purpose, the lysate supernatant was incubated for 1 hr at 4°C with 0.5 ml of Protino Ni‐NTA‐Agarose suspension (Machery & Nagel). The gel beads were then transferred to an open bed chromatography column (Bio‐Rad, Munich, Germany). To remove nonspecifically bound proteins, the column was first eluted trice with 0.5 ml washing buffer containing 10 mM imidazole. Next, the column was washed thrice with 0.5 ml elution buffer 1 containing 25 mM imidazole to elute more tightly bound proteins. Finally, the histidine‐tagged (his‐tag) fusion proteins were eluted rinsing the column seven times with 0.3 ml of elution buffer containing 200 mM imidazole. The majority of the MF‐LOX1 was recovered in the elution fractions 2, 3, and 4.

#### 4.2.1 Eukaryotic expression of MF‐LOX in the baculovirus–insect cell system

To improve the expression yield and the specific activity of the recombinant MF‐LOX, we excised the coding region of the recombinant bacterial expression plasmid using the restriction enzymes *BamHI/HindIII*, purified the digestion product by agarose gel electrophoresis, and inserted the construct into pFastBac HT‐B (Thermo Fisher Scientific). Bacmid and recombinant baculovirus were prepared according to the instructions of the Bac‐to‐Bac® Baculovirus Expression System (Invitrogen Life Technologies/Thermo Fisher). Protein expression was performed using Sf9 cells (ATCC® CRL‐1711) and Insect XPRESS Medium (Biozym, Hessisch Oldendorf, Germany) containing 4 mM glutamine and 0.5% FCS. The 50 ml cell culture (2 x 10^6^ cells/ml) was infected with 5 ml recombinant baculovirus (2nd amplification, MOI of 1) and incubated in Erlenmeyer flasks at 27°C on a shaker platform (120–130 rpm). Cells were harvested by centrifugation (1,500 *g*, 10 min, 4°C) after 72 hr. The cell pellet was resuspended in 2.5 ml PBS containing 2 mM EDTA, sonicated and cell debris was spun down. The lysate supernatant was used for activity assays or further protein purification.

#### 4.2.2 Eukaryotic expression of MF‐LOX1 in HEK293 cells

Since expression of the MF‐LOX1 in Sf9 cells did not improve the specific activity of the final enzyme preparation, we attempted to express the protein in HEK293 cells. For this purpose, the coding sequence of the his‐tag fusion protein construct was excised from the bacterial expression plasmid and inserted into the eukaryotic expression vector pcDNA 3.1(−) using the *XbaI *and *HindIII* restriction sites. Subcloning was performed as described above for cloning from TOPO into pET28b. HEK293 cells were seeded in 6‐well plates (4 x 10^5^ cells/well in 2 ml DMEM [P04‐01550, PAN Biotech GmbH, Aidenbach, Germany, supplemented with 10% FCS]) and grown overnight at 37°C and 5% CO_2_. Plasmid DNA was diluted in Opti‐MEM® I Reduced‐Serum Medium (Gibco/Thermo Fisher) to a final concentration of 2 µg/194 µl. A 6 µl TransIT‐LT1 Transfection Reagent (Mirus Bio LLC, distributed by VWR International GmbH, Darmstadt, Germany) was added and complex formation was allowed for 20–30 min. The complex formed was added to the wells carefully and dropwise. Cells were incubated as described above for 48 hr and were harvested by flushing them off the well bottom with a pipette. After centrifugation and washing, the cells were washed once with PBS, and cells were redissolved in 500 µl PBS containing 2 mM EDTA for activity assay or in sample buffer for SDS‐PAGE.

### 4.3 Site‐directed mutagenesis

Site‐directed mutagenesis was performed using the Pfu Ultra II Hotstart 2XPCR Mastermix (Agilent Technologies, California, USA) as described before (Banthiya et al., 2016). After PCR aliquots of the reactions mixture were transformed into *E. coli *XL1‐Blue competent cells (Thermo Fisher) and plated onto kanamycin agar plates. Five clones were picked and plasmid DNA was prepared using NucleoSpin Plasmid Easy Pure (Macherey‐Nagel). Nucleotide sequencing (Eurofins Genomics, Ebersberg, Germany) confirmed the sequences of the mutant plasmid clones**.**

### 4.4 Fatty acid oxygenase activity assays

Fatty acid oxygenase activity of wild‐type and mutant MF‐LOX1 was determined by HPLC quantification of the reaction products formed during a 3‐min incubation period. For this purpose, aliquots of the MF‐LOX1 preparation were incubated in 0.5 ml of PBS containing different concentrations of fatty acids as substrates. After the incubation period, the hydroperoxy compounds formed were reduced with sodium borohydride and after acidification 0.5 ml of ice‐cold methanol was added. The protein precipitate was spun down and aliquots of the clear supernatant were injected to RP‐HPLC for quantification of the oxygenation products.

### 4.5 HPLC analytics

HPLC analysis of the reaction products was performed on a Shimadzu HPLC system. Reverse phase‐HPLC (RP‐HPLC) was carried out on a Nucleodur C18 Gravity column (Macherey‐Nagel; 250 x 4 mm, 5 μm particle size) coupled with a guard column (8 x 4 mm, 5 μm particle size). A solvent system of methanol/water/acetic acid (85/15/0.1, by volume) was used at a flow rate of 1 ml/min. Peak areas were quantified and the chromatographic scale was calibrated by injecting known amounts of 15‐HETE (7‐point calibration). For more detailed analysis of the chemical structure of the reaction products, normal phase‐HPLC (SP‐HPLC) was performed on a Nucleosil 100‐5 column (250 x 4 mm, 5 μm particle size) with the solvent system *n*‐hexane/2‐propanol/acetic acid (100/2/0.1, by volume) and a flow rate of 1 ml/min. Hydroxylated fatty acid enantiomers were separated and quantified by chiral phase liquid chromatography–mass spectrometry (chiral LC‐MS). The polysaccharide column Amylose‐1 (150 x 2 mm from Phenomenex Aschaffenburg) was kept at 25°C and the products were eluted with a linear gradient of acetonitrile/water/glacial acetic acid (30:70:0.05, v/v/v) to acetonitrile/water/glacial acetic acid (70:30:0.05, v/v/v) over 70 min at a flow rate of 0.2 ml/min. Metabolites were quantified by Single‐Quad‐ESI‐MS (Shimadzu LCMS 2010‐EV) in negative ionization mode.

### 4.6 Activity measurements under normoxic and hyperoxic conditions

To judge the oxygen affinity of MF‐LOX1, we carried out activity assays, in which the oxygen concentration in the reaction mixture was altered. For this purpose, variable volumes of oxygen saturated PBS (hyperoxic) were mixed with argon saturated PBS (anoxic). To obtain the hyperoxic solution, 50 ml PBS was flushed for 3 hr with pure oxygen. Similarly, 50 ml of PBS was flushed with argon to prepare the anoxic medium. Next, a photometric cuvette was filled with argon gas and aliquots of anaerobic PBS (0–0.7 ml) were added under argon atmosphere. The cuvette was closed with a plastic stopper containing two capillary wholes to add additives. Then, different aliquots of hyperoxic PBS (oxygen saturated) were added so that a final reaction volume of 0.7 ml was reached. Next, 10 μL of a partly anaerobized methanolic solution of eicosapentaenoic acid was added and the reaction was started with 50 μL of partially anaerobized enzyme solution.

### 4.7 Membrane oxygenase activity assay

To quantify the membrane oxygenase activity of MF‐LOX1, aliquots of the enzyme preparations were incubated for 15 min in PBS with beef heart submitochondrial particles (1.2 mg membrane protein/ml), which constitute inside‐out vesicles of inner mitochondrial membranes. After the incubation period, the reaction was stopped by the addition of sodium borohydride. Following acidification, the total lipids were extracted from the reaction mixtures (Bligh & Dyer, 1959), the solvent was evaporated, and the remaining ester lipids were reconstituted in 1 ml of a 1:1 mixture of chloroform and methanol. Aliquots of this mixture were hydrolyzed under alkaline conditions. For this purpose, the solvent was evaporated and the remaining lipids were reconstituted in 0.85 ml methanol. 0.15 ml of 40% KOH was added and the ester lipids were hydrolyzed at 60°C for 20 min under argon atmosphere. Then, the samples were cooled down, acidified with 0.15 ml acetic acid and aliquots of this mixture were injected into RP‐HPLC. Following the chromatograms at 235 nm, we quantified the sum of HODE + HETE isomers. To quantify nonoxygenated linoleic acid and nonoxygenated arachidonic acid, we recorded the chromatograms at 210 nm. The chromatographic scale was calibrated by injecting known amounts of 15‐HETE, linoleic acid (LA), and arachidonic acid (AA). Five‐point calibration curves were established for each compound. Finally, we calculated the HODE + HETE/LA + AA ratio, which constitutes a suitable measure for the degree of membrane lipid oxygenation (Aldrovandi et al., 2018; Kuhn et al., 1990).

### 4.8 Determination of the iron content and isoelectric focusing

The iron content of the purified MF‐LOX1 was determined by atom absorbance spectroscopy on a Perkin‐Elmer Life Sciences AA800 instrument equipped with an AS800 autosampler. For calculating the iron load of the enzyme, the iron concentration in the enzyme preparation was related to the protein content.

To determine the native isoelectric point of MF‐LOX1, isoelectric focusing (IF) was carried out. For this purpose, a precasted IF gel (Bio‐Rad, Munich, Germany) was employed and isoelectric focusing was run for 2.5 hr on a Bromma LKB 2197 high‐voltage power supply electrophoresis system. Protein bands were stained with Coomassie blue and the following IF standards were used (phycocyanin, IP 4.45–4.75; β‐lactoglobulin B, IP 5.1; bovine carbonic anhydrase, IP 6.0; human carbonic anhydrase, IP 6.5; equine myoglobin, IP 6.8/7.0; human hemoglobin A, IP 7.1; human hemoglobin C IP 7.5; lentil lectin IP 7.8/8.0/8.2; cytochrome c IP 9.6).

## CONFLICT OF INTEREST

The authors declare that they do not have any conflicts of interest with the content of this article.

## AUTHORS CONTRIBUTION

K.G. expressed MF‐LOX1 in *E. coli* and prepared the recombinant protein from bacterial lysate supernatants and from the lysate of SF9 cells. She also carried out the database searches and characterized the protein‐chemical and enzymatic properties of MF‐LOX1. S.S. expressed the enzyme in SF9 cells. M.B. performed chiral phase LC‐MS. D.H. and H.K. supervised the laboratory work of K.G. and drafted the first manuscript. All co‐authors contributed to manuscript writing. H.K. and D.H. planned the study and coordinated the experiments.

## ETHICS STATEMENT

This paper does not involve any experiments with animals or humans, and thus, there is no ethical issue related to the content of this paper.

## Data Availability

The authors declare that the experimental data published in this paper are made accessible upon request for interested readers.
